# Compatibility and interference of food simulants and organic solvents with the in vitro toxicological assessment of food contact materials

**DOI:** 10.1111/1750-3841.17659

**Published:** 2025-02-04

**Authors:** Athanasios Kourkopoulos, Dick T. H. M. Sijm, Janneke Geerken, Misha F. Vrolijk

**Affiliations:** ^1^ Department of Pharmacology and Toxicology Faculty of Health, Medicine and Life Sciences Maastricht University Maastricht the Netherlands; ^2^ Office for Risk Assessment and Research, Netherlands Food and Consumer Product Safety Authority (NVWA) Utrecht the Netherlands

**Keywords:** food contaminants, in vitro bioassays, organic solvents, plastic food contact materials, safety assessment

## Abstract

Ensuring the safety of food contact materials (FCMs) is paramount, governed by regulations such as Regulation (EC) 1935/2004 and Commission Regulation (European Union [EU]) 10/2011 for plastic FCMs. This study evaluates the compatibility of food simulants specified in the latter regulation with in vitro biological testing. HepG2 and Caco‐2 cell lines were exposed to various concentrations of ethanol and acetic acid. Maximum tolerable amounts of food simulants A, C, and D1 (10%, 20%, and 50% v/v ethanol) were specified at 0.5%, 1.25%, and 2.5%. Food simulant E, Tenax, requires an additional extraction step for the recovery of migrants. An acetone–methanol mixture was selected for its ability to recover both polar and apolar potential migrants. The mixture exerted cytotoxic effects at combined concentrations above 0.5% v/v (0.25% each). Ethanol (Simulants A, C, and D1) interfered with the neutral red uptake (NRU) assay at concentrations above 0.5% v/v, showing no changes in cell viability despite increasing concentrations. Acetic acid (Simulant B) interfered with the NRU assay at 0.5% v/v and lactate dehydrogenase assay at all concentrations, resulting in negative cytotoxicity values due to pH reduction in the exposure medium. Additionally, sample preparation does not interfere with genotoxicity testing, aligning with the European Food Safety Agency (EFSA)’s mandatory testing requirements.

## 1 INTRODUCTION

Ensuring the safety of food contact materials (FCMs) is a critical aspect of food safety. The regulatory framework around the safety of FCMs, particularly Regulation (EC) No 1935/2004, establishes general safety requirements for all FCMs, mandating that materials must not release their constituents into food at levels harmful to human health. Commission Regulation (European Union [EU]) No 10/2011 sets out specific rules for plastic materials and articles intended to come into contact with food. This regulation includes a Union List of authorized substances that can be used in the manufacture of plastic FCMs and sets specific migration limits for these substances to ensure that they do not pose a risk to human health. Additionally, the regulation includes specific migration testing conditions for the assessment of specific migration limits that include food simulants and specific temperature and time conditions.

These food simulants include: ethanol 10% (v/v) (Simulant A) for hydrophilic foods, acetic acid 3% (w/v) (Simulant B) for acidic foods with a pH below 4.5, ethanol 20% (v/v) (Simulant C) for alcoholic foods with up to 20% alcohol content, ethanol 50% (v/v) (Simulant D1) for alcoholic foods with more than 20% alcohol content and oil‐in‐water emulsions, vegetable oil (Simulant D2) for foods containing free fats at the surface, and poly(2,6‐diphenyl‐*p*‐phenylene oxide) (PPPO) commercially known as Tenax (Simulant E) for dry foods. Those food simulants aim to represent as accurately as possible the realistic migration occurring through the interaction between the FCM and food under foreseen intended usage conditions. Additionally, various other solvents and food simulants have been used in literature for the preparation of samples, including 95% v/v ethanol, isooctane, methanol, *n*‐heptane, and ethane (Bradley et al., 2008, 2010; Groh & Muncke, 2017).

In vitro bioassays play a pivotal role in evaluating the potential toxicity of substances that may migrate from FCMs into food. Target tissues such as liver and the intestinal epithelium are primary targets for food contaminants (Eskola et al., 2019; Pinton & Oswald, 2014). Chemicals used in the FCMs in the EU are required to be assessed for their mutagenic and genotoxic potential under the European Food Safety Authority's (EFSA) guidelines involving the in vitro bacterial reverse mutation assay (Ames test) and in vitro micronucleus assays (EFSA, 2016).

The sample preparation for the bioassays relies on food simulants and extraction solvents for the recovery of food contact chemicals from the FCMs. Organic solvents, including ethanol and acetic acid, can exert cytotoxic effects to the biological systems used for the investigation as demonstrated by various studies (Pivatto et al., 2020; Tanneberger et al., 2010; Timm et al., 2013). Therefore, these can interfere with the measurements via the assay resulting in misleading assessments and interpretations.

This article explores the complex application of food simulants and extraction solvents in in vitro bioassays, highlighting both their compatibility and the challenges they present in ensuring the safety of FCMs. The investigation emphasizes conventional bioassays used to evaluate cytotoxic potency on HepG2 and Caco‐2 cell lines, which are commonly employed for assessing hepatotoxicity and colon toxicity. It also examines the bacterial reverse mutation assay (Ames test) and the in vitro micronucleus assay, both required by the EFSA for evaluating the mutagenic and genotoxic potential of food contact chemicals. The novelty of this study lies in its comprehensive analysis of the compatibility and limitations of organic solvents in regulatory food simulants and their impact on the performance and efficiency of in vitro biological screening. This includes both conventional cytotoxicity and cell viability assays and regulatory mutagenicity and genotoxicity assays. The research ultimately aims to provide a thorough understanding of the factors affecting the effectiveness of in vitro bioassays in food safety assessments, emphasizing the importance of direct involvement of food simulants without additional handling steps that could lead to the loss of relevant toxicants.

## 2 MATERIALS AND METHODS

### 2.1 Materials

The cell culture materials included Dulbecco's Minimum Eagle Medium (DMEM) with 4.5 g/L glucose, phenol red, and GlutaMAX, sourced from Gibco (Cat. No. 61965059). This medium was enhanced with 100 U/mL penicillin and 100 µg/mL streptomycin, also from Gibco (Cat. No. 15140122), for all cell lines. The exposure medium was DMEM (4.5 g/L glucose, phenol red‐free) from Gibco (Cat. No. 31053028), supplemented with 10% (v/v) fetal bovine serum (Gibco, Cat. No. A5256701), 100 U/mL penicillin, 100 µg/mL streptomycin, and 2 mM l‐glutamine (Thermo Fisher Scientific, Cat. No. 25030024). Additional reagents included Dulbecco's Phosphate Buffered Saline (DPBS) (Gibco, Cat. No. 14190144).

For the preparation of food simulants and organic solvents for testing, the materials included acetic acid (VWR Chemicals, Cat. No. 20102.292), ethanol (Merck, Cat. No. 1.00983.2500), anhydrous methyl alcohol (Macron Fine Chemicals, Cat. No. 3004‐25), acetone (Sigma Aldrich, Cat. No. 179124), acetonitrile (Supelco, Cat. No. 100665), dimethylsulfoxide (DMSO) (Sigma Aldrich, Cat. No. D5879), dimethylformamide (DMF) (Sigma Aldrich, Cat. No. D4551), tetrahydrofuran (THF) (Sigma Aldrich, Cat. No. T3125), and dioxane (Supelco, Cat. No. 103132).

For the 3‐(4,5‐dimethylthiazol‐2‐yl)‐2,5‐diphenyltetrazolium bromide (MTT) assay, the materials included MTT (Sigma Aldrich, Cat. No. 1117140001), Triton X‐100 (Merck, Cat. No. X100), and DMSO (Sigma Aldrich, Cat. No. D8418). The lactate dehydrogenase (LDH) assay materials comprised 2× LDH assay buffer, 2‐(4‐iodophenyl)‐3‐(4‐nitrophenyl)‐5‐phenyltetrazolium chloride 95% (INT) (VWR, Cat. No. B22301.03), lactic acid (VWR, Cat. No. 20366.293), *N*‐methylphenazonium methyl sulfate (Merck, Cat. No. P9625), and nicotinamide adenine dinucleotide (Sigma Aldrich, Cat. No. 1.24542). For the neutral red uptake (NRU) assay, neutral red (3‐amino‐7‐dimethylamino‐2‐methyl‐phenazine hydrochloride) (Cat. No. N4638) was obtained from Sigma Aldrich.

For the bacterial reverse mutation assay (Ames test), the materials included mutated *Salmonella typhimurium* stocks from Trinova: TA 98 (Cat. No. 71‐098L), TA 100 (Cat. No. 71‐100L), TA 102 (Cat. No. 71‐102L), TA 1535 (Cat. No. 71‐1535L), and TA 1537 (Cat. No. 71‐1537L). Additional materials included 90 mm sterile Petri dishes (Thermo Fisher Scientific, Cat. No. 101IRR), bacteriological agar (Sigma Aldrich, Cat. No. A5306), d‐biotin (Novabiochem, Cat. No. 8512090005), Nutrient Broth No. 2 (Dehydrated) (Thermo Fisher Scientific, Cat. No. CM0067B), d‐glucose (Sigma Aldrich, Cat. No. G8270), 4‐nitro‐*o*‐phenylenediamine (NPD) (Sigma Aldrich, Cat. No. 108898), 2‐aminoanthracene (2AA) (Sigma Aldrich, Cat. No. A38800), 2‐aminofluorene (2AF) (Sigma Aldrich, Cat. No. A55500), tetracycline (Sigma Aldrich, Cat. No. T3258), sodium azide (SA) (Sigma Aldrich, Cat. No. 1066880100), crystal violet (Sigma Aldrich, Cat. No. C0775), and ampicillin (Thermo Fisher Scientific, Cat. No. J66972.AB).

For the in vitro micronucleus assay, the materials included Cytochalasin B (Sigma Aldrich, Cat. No. C2743‐200UL), cyclophosphamide monohydrate (Merck, Cat. No. 93813‐100MG), Mitomycin C (Mit C) (Sigma Aldrich, Cat. No. 10107409001), colchicine (Carl Roth, Cat. No. 8884.1), and GelRed nucleic acid stain (Merck, Cat. No. SCT123).

### 2.2 Cell culture procedures

The experiments utilized several cell lines: the human hepatocyte cell line (HepG2) and the human colon epithelial cell line (Caco‐2). The cells were cultured in the specified culture medium (as detailed in the materials section). Cells were subcultured under sterile conditions to 80%–90% confluence at 37°C and 5% CO_2_ and detached using 0.25% v/v trypsin‐EDTA phenol red solution. The passage number was kept below 30 for HepG2 and between 20 and 40 for Caco‐2 cell lines.

### 2.3 3‐(4,5‐Dimethylthiazol‐2‐yl)‐2,5‐diphenyltetrazolium (MTT) assay

HepG2 and Caco‐2 cells were seeded in 96‐well plates at a density of 25,000 cells per well in culture medium. The plates were then incubated for 24 h at 37°C with 5% CO_2_. After incubation, the seeding medium was removed from each well, and the cells were exposed for 24 h to diluted extracts in exposure medium (as specified in the materials section). Following exposure, the medium was aspirated from each well, and 100 µL of 1 µg/mL MTT solution in DPBS was added to each well. The plates were incubated for 1 h at 37°C with 5% CO_2_. After this incubation, the MTT solution was removed, and 100 µL of DMSO was added to each well. The plates were gently shaken on an orbital plate shaker for 30 min. Finally, the optical density (absorbance) was measured at 540 nm using a SpectraMax iD3 plate reader (Molecular Devices). A 1% v/v Triton X‐100 solution in exposure medium served as a reference control.

### 2.4 Lactate dehydrogenase (LDH) assay

HepG2 and Caco‐2 cells were seeded and exposed in the same manner as described for the MTT assay. After 24 h of exposure, 50 µL of the exposure medium was collected from each well into another 96‐well plate for the LDH assay. The procedure followed the steps and materials outlined by Chan et al. (2013). Briefly, 50 µL of 2× LDH assay buffer was added to each well containing 50 µL of the collected exposure medium. The plates were incubated for 30 min at 25°C, and the reaction was stopped by adding 50 µL of 1 M acetic acid solution to each well. The plates were then measured at 510 nm using a SpectraMax iD3 plate reader. A 1% v/v Triton X‐100 solution in exposure medium was used as a reference control.

### 2.5 Neutral red uptake (NRU) assay

The NRU assay was performed based on the protocol by Repetto et al. (2008) with small adjustments. In brief, HepG2 and Caco‐2 cell lines were seeded and exposed to organic solvents in the same manner as detailed for the MTT assay. Post‐exposure, the cells were examined using an optical microscope. The exposure medium was then removed, and the cells were washed twice with 100 µL of DPBS. Subsequently, 100 µL of neutral red solution in DPBS was added to each well, and the plates were incubated for 2 h at 37°C with 5% CO_2_. The neutral red solution was aspirated, and the wells were washed twice with 100 µL of DPBS. Finally, 150 µL of neutral red destain solution (comprising 50% ethanol, 49% deionized water, and 1% v/v glacial acetic acid) was added to each well. The plates were shaken rapidly for 10 min on an orbital plate shaker, and the optical density of the neutral red extract was measured at 540 nm using a SpectraMax iD3 plate reader.

### 2.6 Bacterial reverse mutation assay (Ames test)

The bacterial reverse mutation assay was conducted following the guidelines in Organization for Economic Co‐operation and Development's (OECD) Technical Guideline 471. The mutated Salmonella test strains used were TA98, TA100, TA102, TA1535, and TA1537. The test was performed both with and without S9 mix.

The bacterial strains were inoculated from frozen stocks the day before the experiment and cultured overnight in Nutrient Broth Oxoid No. 2 in a sterile Schott flask at 37°C. The following day, the strains were shaken on an orbital plate shaker at 200 rpm for 4 h. Minimal agar plates were prepared according to Maron and Ames (1983). The viability and number of bacterial cells were determined via trypan blue staining and light microscopy counting. A volume of 0.1 mL of 10^8^ viable cells were mixed with 0.5 mL of sterile buffer or 10% v/v S9 mix solution in PBS containing the diluted migration sample or extract to be tested. The dilution was adjusted to noncytotoxic levels based on preliminary exposure tests. Two mL of overlay agar was added to the 0.6 mL solution, and the mixture was poured onto prepared minimal agar plates and swirled gently to cover the surface. The plates were left to dry and then incubated at 37°C for 48 h. Following treatment, the developed colonies were counted manually.

Phenotype control plates were prepared for the exposed strains, treated with 8 mg/mL ampicillin, 0.5 mg/mL tetracycline, and 1 mg/mL crystal violet. Additionally, the plate was covered in aluminum foil with a small part exposed to UV light. Measurements were carried out in triplicates over 3 separate days.

In the absence of S9 mix, 20 µg/plate of NPD was used as a reference mutagenic compound for strains TA98, TA100, and TA1537. For strain TA102, 0.5 µg/plate of Mit C was used as a reference control, whereas 2 µg/plate of SA was used for strain TA1535. In the presence of S9 mix, 10 µg/plate of 2AF was used as a reference compound for strains TA98, TA100, and TA102, and 2 µg/plate of 2AA was used for strains TA1535 and TA1537.

### 2.7 In vitro micronucleus assay

The in vitro micronucleus assay was conducted using HepG2 and Caco‐2 cell lines according to OECD TG 487. Briefly, cells were seeded in a 6‐well plate and incubated overnight at 37°C with 5% CO_2_. The culture medium was then replaced with phenol red‐free medium containing noncytotoxic concentrations of the migration samples and extracts. The treatment involved three separate exposures. The first exposure lasted 4 h, followed by replacement with medium containing 4.5 µg/mL Cytochalasin B. The second exposure followed the same schedule but included 1% v/v S9 mix in the medium. The third exposure lasted 48 h with medium supplemented with 4.5 µg/mL Cytochalasin B. After treatment, the cells were washed twice with PBS, detached using 0.25% v/v trypsin‐EDTA phenol red solution, neutralized with culture medium, and centrifuged. The cells underwent hypotonic treatment as outlined by Heger et al. (2018), were fixed on glass slides, and stained with GelRed nucleic acid stain. The slides were imaged at 10× magnification using BioTek's Cytation 3 Cell Imaging Multi‐Mode Reader. The number of micronuclei per 2000 nuclei was manually counted.

Samples were tested both with and without S9 mix. The procedure was carried out in duplicate over 2 separate days. Reference compounds included 0.5 µg/mL Mit C for the 4‐h treatment without S9 mix, 10 µg/mL cyclophosphamide for the 4‐h treatment with S9 mix, and 0.5 µg/mL colchicine for the 48‐h exposure.

### 2.8 Statistical analysis

The no‐observed‐adverse‐effect‐concentrations (NOAECs) for the migration samples and extracts were identified by comparing untreated and treated samples using one‐way analysis of variance with a *p*‐value threshold of 0.05. This analysis was followed by Tukey's honestly significant difference post hoc test. The normality of residuals was assessed using the Shapiro–Wilk test, and the homogeneity of variances was evaluated with Levene's test.

## 3 RESULTS AND DISCUSSION

### 3.1 Tolerance of HepG2 and Caco‐2 cell lines to food simulants and interference with the assays

The organic solvents specified in Commission Regulation (EU) 10/2011 were assessed using the MTT, NRU, and LDH assays to determine their impact on the viability of HepG2 and Caco‐2 cell lines. The highest concentration at which ethanol and acetic acid did not exert adverse effects was determined using NOAEC values. These NOAEC values were then used to establish food simulant concentrations at which no background effects are observed via the bioassays. The goal is to eliminate background effects on the biological systems used to investigate the toxicity of FCMs due to the involvement of organic solvents in the preparation of FCM samples.

#### 3.1.1 Food simulants A, C, and D1 (10%, 20%, and 50% v/v ethanol)

**FIGURE 1:**
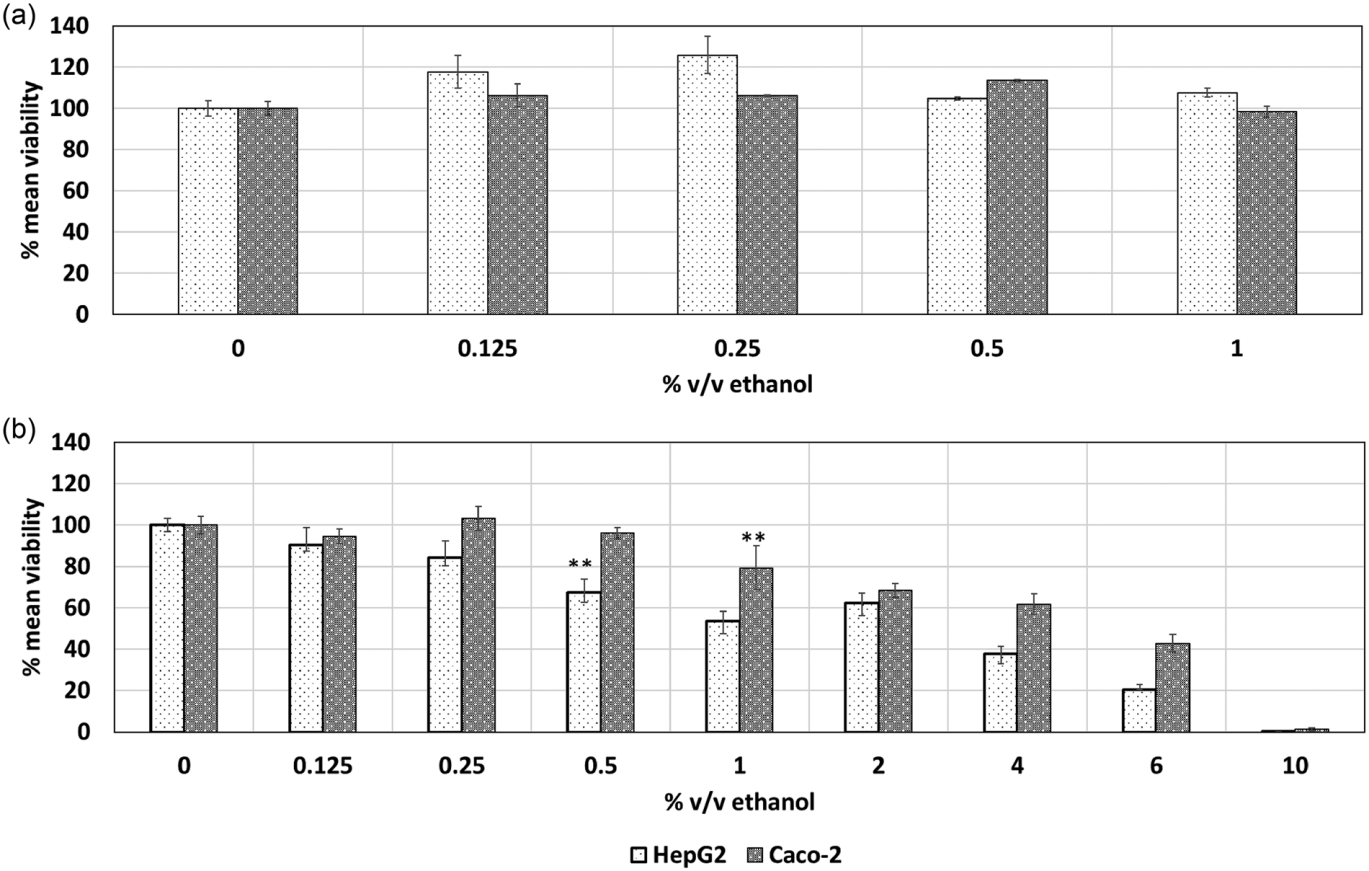
Effect of ethanol on the mean viability of HepG2 and Caco‐2 cell lines following 24 h of exposure as determined via the (a) neutral red uptake (NRU) and (b) MTT assays. Data are shown as mean + standard error of the mean (SEM) (*n* = 9). Differences were considered to be statistically significant when *p < 0.05; **p < 0.01; ***p < 0.001.

The NRU assay (Figure 1a) showed no decrease in cell viability with increasing ethanol concentrations up to 2% v/v. However, the MTT assay (Figure 1b) revealed a gradual decrease in the viability of HepG2 and Caco‐2 cell lines with increasing ethanol concentrations. Exposure to 0.5% v/v ethanol significantly decreased HepG2 cell viability by 32.6%, whereas 1% v/v ethanol reduced Caco‐2 cell viability by 20%.

**FIGURE 2:**
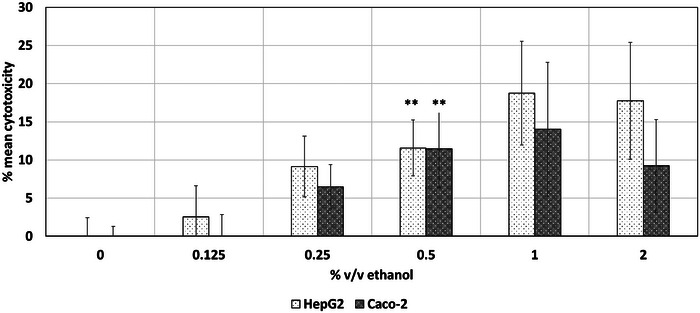
Mean cytotoxic effect of ethanol to HepG2 and Caco‐2 cell lines following 24 h of exposure as determined via the lactate dehydrogenase (LDH) assay. Data are shown as mean + standard error of the mean (SEM) (*n* = 9). Differences were considered to be statistically significant when *p < 0.05; **p < 0.01; ***p < 0.001.

Increasing ethanol concentrations exerted a cytotoxic effect on HepG2 and Caco‐2 cell lines, as shown in Figure 2. The cytotoxicity was proportional to the ethanol concentration in a concentration‐response manner, with significant cytotoxic potential observed at concentrations above 0.5% for both cell lines.

Based on the obtained results on cell viability and cytotoxicity, a NOAEC value of 0.25% v/v ethanol for HepG2 cell lines was identified for HepG2 cell line as introduced in Table 1. A NOAEC of 0.5% v/v ethanol was determined via MTT for Caco‐2 cell line, whereas a value of 0.25% was derived from the LDH assay. The NRU assay did not show any changes in the viability of HepG2 and Caco‐2 cell lines as a result of their exposure to ethanol (Figure 1a). Therefore, threshold values could not be derived via this assay. Ethanol as such seems to interfere with the function of the NRU assay. Tapani et al. (1996) observed an increasing cytotoxic effect of ethanol with increasing concentrations. Their study conducted cytotoxicity experiments on three malignant rat hepatocyte cell lines, exposing them to ethanol concentrations ranging from 0% to 50% v/v for 0.25–60 min. A proportional exposure time‐dependent effect was identified with regard to cytotoxicity, assessed using the trypan blue exclusion test. Similar to the responses observed in our study, the authors reported no significant differences in ethanol tolerance among the cell types.

**TABLE 1:** Summary of no‐observed‐adverse‐effect‐concentrations (NOAECs) for exposed HepG2 and Caco‐2 cell lines to ethanol as determined via the MTT, lactate dehydrogenase (LDH), and neutral red uptake (NRU) assays.

NOAEC (% v/v)	HepG2	Caco‐2
MTT	0.25%	0.5%
NRU	N/A	N/A
LDH	0.25%	0.25%

Low concentrations of ethanol around 1 mM have been demonstrated to inhibit cell proliferation and induce apoptosis via Caspase‐3 activation, whereas higher concentrations close to 10 mM can lead to increased uncontrolled cell death and further inhibit proliferation in the HepG2 cell line (Castañeda & Kinne, 2000). In this study, the cytotoxic effect of ethanol was compared between HepG2 and rat hepatocytes, the latter exhibiting higher resilience to increasing concentrations of ethanol from 1 to 10 mM.

Nguyen et al. (2020) reported significant inhibition of cell proliferation at ethanol concentrations above 2.5% v/v. For the Caco‐2 cell line, various concentration and time‐dependent effects are reported. Park et al. (2012) reported a LOAEC of 7% v/v following a 1‐h incubation, as measured via the MTT assay, whereas Asai et al. (2003) demonstrated an increase in apoptosis markers at 5% v/v ethanol concentration following 3 h of exposure. Therefore, it is plausible that the longer the exposure of the Caco‐2 cell line to ethanol, the lower the harmful concentration threshold. In this case, the 24‐h exposure yielded the cytotoxicity threshold presented in Table 1.

#### 3.1.2 Food simulant B (3% v/v acetic acid)

**FIGURE 3:**
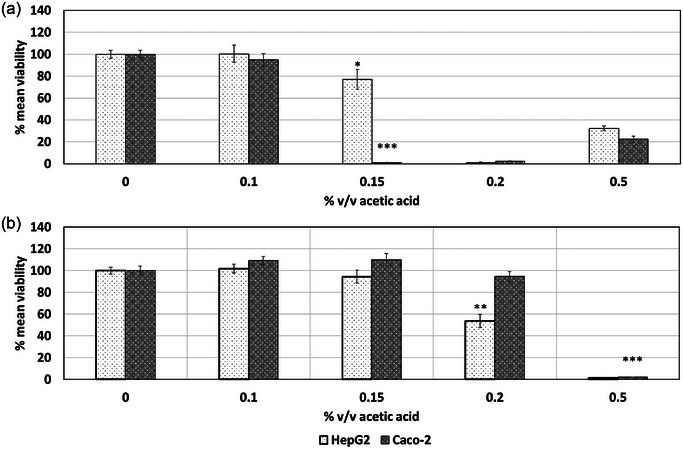
Effect of acetic acid on the mean viability of HepG2 and Caco‐2 cell lines following 24 h of exposure as determined via the (a) neutral red uptake (NRU) and (b) MTT assay. Data are shown as mean + standard error of the mean (SEM) (*n* = 9). Differences were considered to be statistically significant when *p < 0.05; **p < 0.01; ***p < 0.001.

Acetic acid led to a decrease in the viability of both HepG2 and Caco‐2 cells as assessed via the NRU and MTT assays (Figure 3a,b). Significant decrease in the mean viability of both cell lines was observed at 0.15% v/v acetic acid in culture medium as presented in Figure 3a via the NRU assay. HepG2 presented a decrease of 22% in their mean viability, whereas the decrease was more pronounced for Caco‐2 cell line which presented a total loss of viability at 0.15% v/v acetic acid concentration. A small increase of cell viability was observed for both cell lines at 0.5% v/v acetic acid concentration. However, this does not follow the concentration‐dependent manner. Similarly to the NRU, exposure of HepG2 and Caco‐2 cell lines to increasing concentrations of acetic acid resulted in decrease of the viability of those cells in a concentration‐dependent manner as determined via the MTT assay as shown in Figure 3b. HepG2 presented a significant decrease of 46.2% in their mean viability in response to exposure to 0.2% v/v acetic acid, whereas a total loss of viability was observed for Caco‐2 cells at 0.5% v/v acetic acid concentration. In Figure 2a, an increase in the response of the NRU assay is observed at a 0.5% v/v acetic acid concentration, following a complete absence of cell viability for both HepG2 and Caco‐2 cell lines at a 0.2% v/v concentration. Additionally, an increase in the response of the NRU assay is observed at 0.5% v/v acetic acid concentration following complete absence of cell viability for both HepG2 and Caco‐2 cell lines at 0.2% v/v concentration. The NRU assay indeed relies on the ability of viable cells to incorporate and bind the neutral red dye within lysosomes. At physiological pH, the dye is neutral and can easily penetrate cell membranes. However, within the acidic environment of the lysosomes, the dye becomes positively charged and is retained. To this extent, it is plausible that at 0.5% v/v acetic acid exposure, the acetic acid may have accumulated in the cell structures and thus altered intracellular pH, leading to a retention of the dye in cell compartments other than lysosomes or the cytoplasm. The destain solution outlined in Repetto et al. (2008), containing 50% ethanol, 49% deionized water, and 1% glacial acetic acid, is indicative of the affinity of the dye for low pH and acetic acid. This could result in an apparent increase in cell viability as measured by the NRU assay.

Acetic acid interfered with the function of the LDH assay showing negative cytotoxicity values. Therefore, the assay is not considered suitable for the assessment of samples prepared with food simulant A. NOAEC values of 0.15% v/v acetic acid were determined for HepG2 via both MTT and NRU assays. In addition, NOAEC values of 0.15% and 0.2% v/v acetic acid were determined for Caco‐2 cell lines via MTT and NRU assays, respectively, as summarized in Table 2. Lestari et al. (2005) reported a low NOAEC threshold for acetic acid tolerance of HepG2 cell line at 0.17 mM via the MTS assay following 4 h of exposure.

**TABLE 2:** Summary of no‐observed‐adverse‐effect‐concentrations (NOAECs) for exposed HepG2 and Caco‐2 cell lines to acetic acid as determined via the MTT, lactate dehydrogenase (LDH), and neutral red uptake (NRU) assays.

NOAEC (% v/v)	HepG2	Caco‐2
MTT	0.15%	0.15%
NRU	0.15%	0.2%
LDH	N/A	N/A

### 3.2 Tolerance of used cell lines to extraction solvents—food simulant E (PPPO; 50%/50% v/v acetone–methanol)

The preparation of suitable samples for in vitro biological investigation with food simulant E should involve an additional preparation step aiming to the recovery of the chemicals from the food simulant. Food simulant E, PPPO, is in the form of an insoluble powder, and, as such, it does not allow for the homogeneous dissolution in exposure medium. The simulant can be additionally extracted for the recovery of the migrating chemicals in a solvent that is compatible with the in vitro biological assays to be conducted. The solvents involved in the preparation should be miscible with water in order to be capable of forming a homogeneous solution involved in the exposure of the biological system and should not pose background adverse effect to the system during the investigation of the migrant chemicals toxicity.

The investigation focused on the selection of a combination of solvents with different polarities that are water miscible and miscible with one another. The combination of solvents with distinct polarity aims to recover holistically the polar and apolar potential migrants that might be present in PPPO. Additionally, the use of such combination of solvents must allow for the formation of a homogeneous exposure medium and, at feasible concentrations, should not exert background adverse effects to the biological system used for the investigation or the bioassays involved.

Table 3 introduces water‐miscible organic solvents classified based on their relative polarity compared to water. The solvents were distributed to three categories of 0–0.2, 0.2–0.5, and 0.5–0.8 relative polarity compared to water. The solvents belonging to the first category, dioxane and THF, were screened preliminarily and discarded from further involvement in the investigation due to their high cytotoxic potential to HepG2 and Caco‐2 cell lines.

**TABLE 3:** Water‐miscible solvents classification based on their relative polarity compared to water.

Classification ranges	0–0.2	0.2–0.5	0.5–0.8
**Solvent (relative polarity to water)**		Tetrahydrofuran (THF) (0.207) Acetone (0.355)	2‐Propanol (0.546)
	Dioxane (0.164)	Dimethylformamide (DMF) (0.386)	Ethanol (0.654)
		Dimethylsulfoxide (DMSO) (0.444)	Acetic acid (0.648)
		Acetonitrile (0.46)	Methanol (0.762)

*Source*: Reichardt and Welton (2010).

**FIGURE 4:**
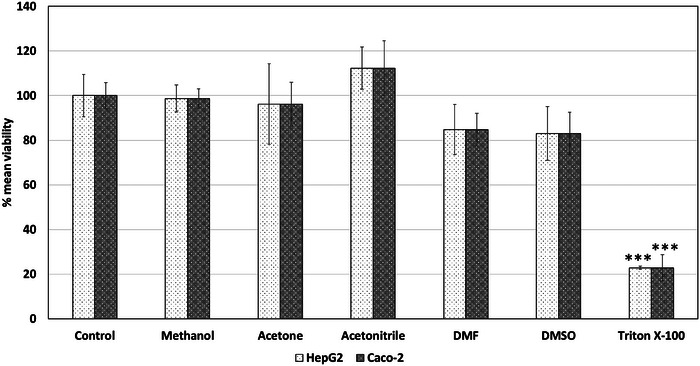
Effect of organic solvents at 0.5% v/v individual concentration on the mean viability of exposed HepG2 and Caco‐2 cell lines for 24 h exposure as determined via MTT. Data are shown as mean + standard error of the mean (SEM) (*n* = 9). Differences were considered to be statistically significant when *p < 0.05; **p < 0.01; ***p < 0.001.

Acetone, acetonitrile, DMF, and DMSO were investigated as potential apolar solvents, whereas methanol was investigated as a possible suitable polar solvent. The effects of these solvents at 0.5% v/v concentration in exposure medium were screened via MTT for HepG2 and Caco‐2 cell lines as introduced in Figure 4. Methanol, acetone, and acetonitrile did not present a notable decrease in the viability of the two aforementioned cell lines at 0.5% v/v concentration, whereas DMF and DMSO presented a borderline non‐statistically significant reduction (*p*‐value slightly above 0.05) in the viability of HepG2 and Caco‐2 cell lines.

**FIGURE 5:**
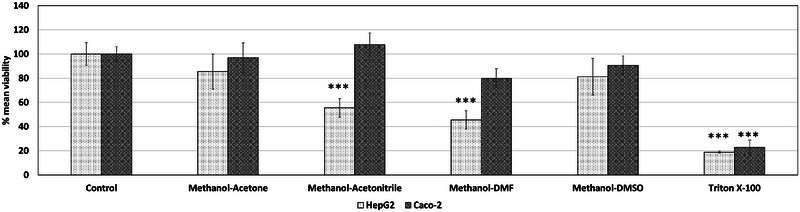
Effect of organic solvents mixture at 0.5% v/v total organic concentration (0.25% and 0.25% v/v individual concentration) on the mean viability of exposed HepG2 and Caco‐2 for 24 h exposure as determined via MTT. Data are shown as mean + standard error of the mean (SEM) (*n* = 9). Differences were considered to be statistically significant when *p < 0.05; **p < 0.01; ***p < 0.001.

In addition to the effects of the individual solvents, the combination of methanol with acetone, acetonitrile, DMF, and/or DMSO was investigated for combined toxicity in HepG2 and Caco‐2 cells (Figure 5). Methanol and acetone at a combined concentration of 0.5% v/v (0.25% each) did not significantly decrease cell viability. Similarly, methanol and DMSO did not exert statistically notable adverse effects after 24 h of exposure. However, methanol combined with acetonitrile and DMF reduced HepG2 viability by 45.5% and 55.4%, respectively. The combination of methanol and acetone was preferred over methanol and DMSO, as acetone is less polar than DMSO, covering a wider range of apolar chemicals predicted to be present in FCMs and PPPO.

**FIGURE 6:**
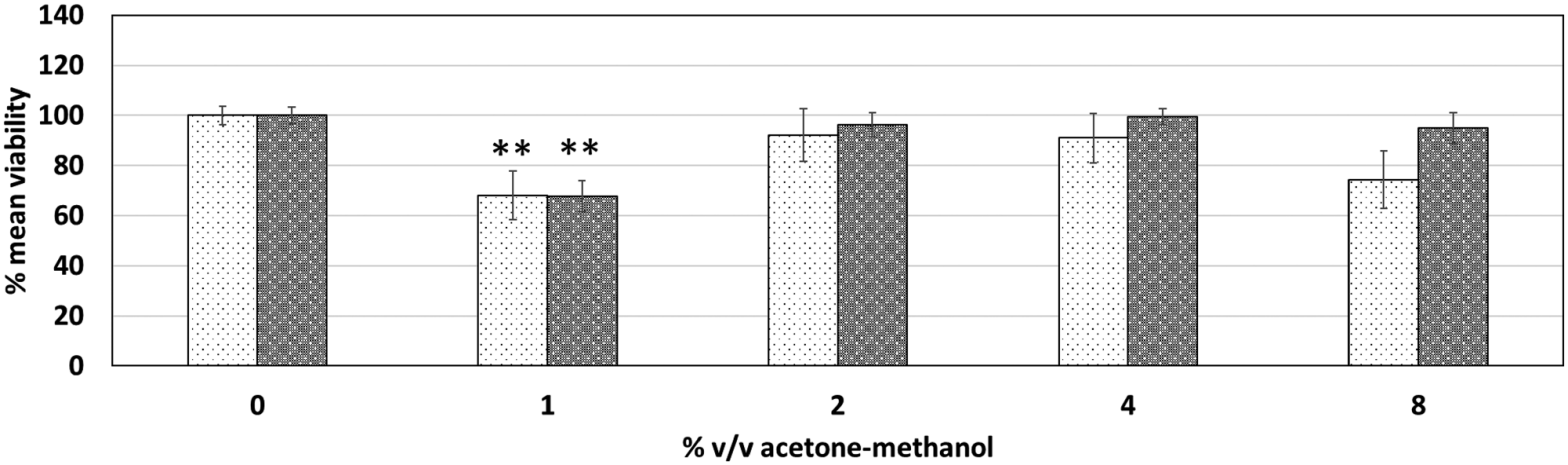
Effect of acetone and methanol on the mean viability of HepG2 and Caco‐2 cells as determined via neutral red uptake (NRU) following 24 h exposure. The concentration is expressed in % v/v total organic content (individual solvent concentration is half of the total organic content). Data are shown as mean + standard error of the mean (SEM) (*n* = 9). Differences were considered to be statistically significant when *p < 0.05; **p < 0.01; ***p < 0.001.

**FIGURE 7:**
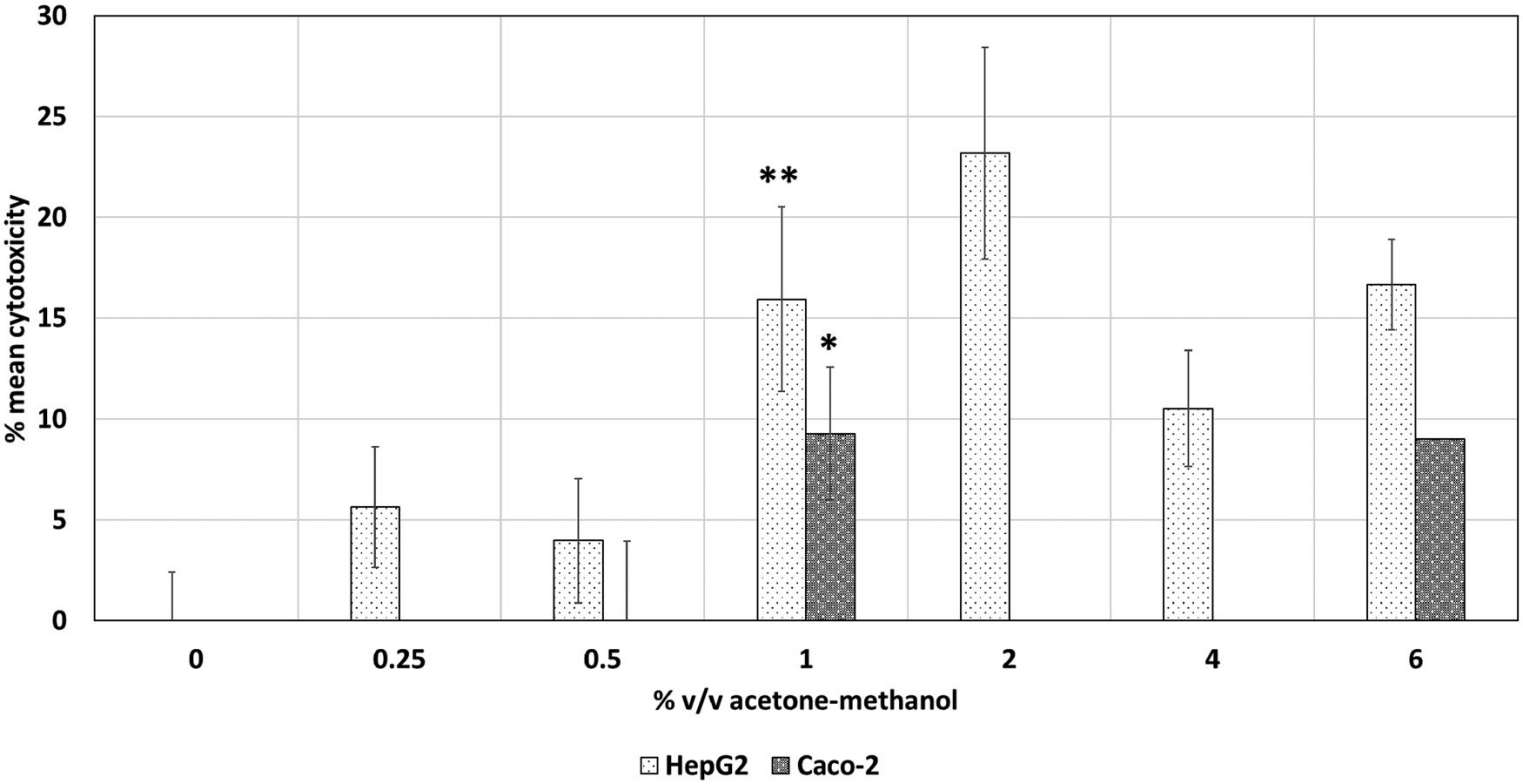
Cytotoxic effect of acetone and methanol on exposed HepG2 and Caco‐2 cell lines as assessed via the lactate dehydrogenase (LDH) assay following 24 h exposure. The concentration is expressed in % v/v total organic content (individual solvent concentration is half of the total organic content). Data are shown as mean + standard error of the mean (SEM) (*n* = 9). Differences were considered to be statistically significant when *p < 0.05; **p < 0.01; ***p < 0.001.

After selection of acetone and methanol as the preferred combination, it was aimed to identify its NOAEC using the NRU and LDH assay. The acetone and methanol mixture at a combined concentration of 1% v/v significantly reduced the mean viability of HepG2 and Caco‐2 cell lines by 32% in the NRU assay, as shown in Figure 6 and Figure 7. This exposure also led to a statistically significant increase in cytotoxicity of 15.9% for HepG2 and 9.3% for Caco‐2 cell lines. Consequently, a NOAEC of 0.5% v/v total combined content (0.25% v/v individual solvent concentration) was determined via both the NRU and LDH assays.

Table 4 presents the tolerable concentrations of HepG2 and Caco‐2 cell lines for the food simulants. These threshold values were calculated based on the determined NOAECs for the organic solvents involved in the food simulants. The lowest determined NOAECs were used for this calculation, as defined via the NRU, MTT, and LDH assays for the two cell lines. Additionally, the sample dilution that follows the involvement of such concentrations in biological testing is provided.

**TABLE 4:** Recalculation of no‐observed‐adverse‐effect‐concentrations (NOAECs) to food simulant tolerance in exposure medium.

Migration	Solvent	% v/v NOAEC/% v/v food simulant in exposure medium	x sample dilution
Food simulant A	10% v/v ethanol	0.25/2.5	40
Food simulant B	3% v/v acetic acid	0.15/5	20
Food simulant C	20% v/v ethanol	0.25/1.25	80
Food simulant D1	50% v/v ethanol	0.25/0.5	200
Food simulant E	Poly(2,6‐diphenyl‐p‐phenylene oxide), particle size 60–80 mesh, pore size 200 nm (PPPO) extracted with 1:1 methanol‐acetone	0.5/0.5	200

In summary, the food simulants specified in Commission Regulation (EU) 10/2011 can be efficiently used for preparing FCM samples for in vitro biological testing. The concentration of the food simulants involved in the assessment should be controlled to avoid background effects and interference with the screening. Additionally, current data show that not all assays are capable of reliably assessing FCMs prepared with the food simulants. Therefore, sample preparation conditions and assay selection should be based on the specific requirements of each procedure, as suggested by Groh and Muncke (2017). NOAEC values of 0.25% v/v, 0.15% v/v, and 0.5% v/v were determined for ethanol, acetic acid, and the acetone–methanol mixture, respectively. In this context, Nguyen et al. (2020) reported that ethanol and methanol concentrations ranging from 0.15% to 2.5% did not inhibit cell proliferation of HepG2 and three other cell lines following 24 h of exposure. This study proposes using these two solvents as better alternatives to DMSO, aligning with the observations derived in this study. Therefore, these solvents at the proposed concentrations do not interfere with the homeostasis of the biological systems under investigation.

Food simulant B, involving 3% v/v acetic acid, is not compatible with cytotoxicity assessment via the LDH assay. This incompatibility might be due to a loss of LDH functionality caused by the reduction of the pH of the exposure medium by acetic acid, subsequently used for the LDH assay. Additionally, cell viability assessment via NRU cannot be performed for acetic acid concentrations above 0.5% v/v, as suggested by the artificial increase in cell viability observed for the two cell lines. Furthermore, the NRU assay is unsuitable for assessing samples prepared with food simulants A and D1, involving 10% and 50% v/v ethanol. The assay did not show any changes in cell viability related to increasing ethanol concentration, whereas the MTT and LDH assays did present a decrease in viability and an increase in observed cytotoxicity, respectively. The combination of methanol and acetone is compatible for preparing samples via the extraction of food simulant E. The solvents did not show background cytotoxicity responses at a 0.5% v/v combined concentration and can create a homogeneous exposure sample.

### 3.3 Compatibility of food simulants with mandatory genotoxicity tests required by the EFSA

The EFSA guidelines involve the Ames test and the in vitro micronucleus assay as tools for the mandatory assessment of the genotoxic potential of food contact chemicals. The food simulants and organic solvents involved as vehicles in the analysis should not interfere with the procedure and, therefore, influence the outcome of the analysis.

#### 3.3.1 Bacterial reverse mutation assay (Ames test)

**FIGURE 8:**
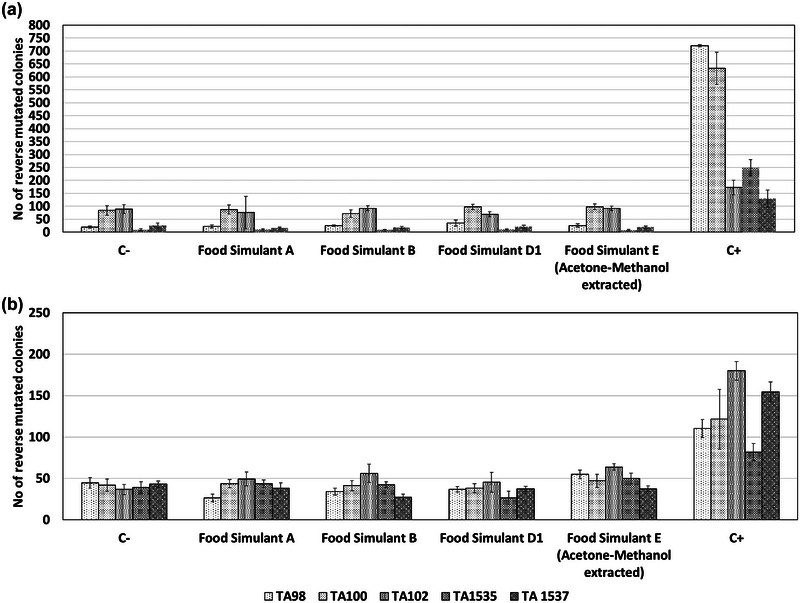
Effect of food simulants on the bacterial reverse mutation assay in the absence (a) and presence (b) of rat liver S9 mix. Data are shown as mean + standard error of the mean (SEM) (*n* = 9). Differences were considered to be statistically significant when *p < 0.05; **p < 0.01; ***p < 0.001.

Food simulants A, B, D1, and the combination of acetone and methanol used for preparing samples from simulant E were tested via the bacterial reverse mutation assay for background effects. The *S. typhimurium* strains used for the assay were TA98, TA100, TA102, TA1535, and TA1537, both in the presence and absence of an external metabolizing system (rat liver S9), as mandated by OECD technical guideline 471. The involvement of food simulants at the concentrations specified in Table 3 did not present any background effects or interference with the assay's function, neither in the presence nor absence of the microsomes, as shown in Figure 8a,b.

#### 3.3.2 In vitro micronucleus assay

**FIGURE 9:**
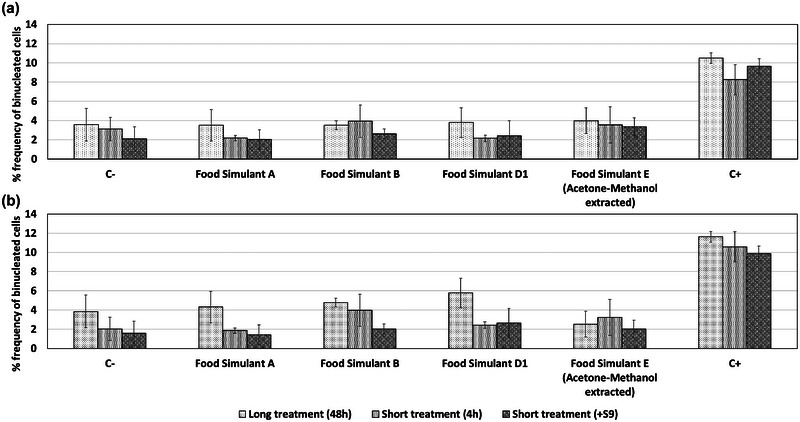
Effect of food simulants and organic solvents on the frequency of binucleated exposed HepG2 (a) and Caco‐2 (b) cell lines as assessed via the in vitro micronucleus assay. Data are shown as mean + standard error of the mean (SEM) (*n* = 9). Differences were considered to be statistically significant when *p < 0.05; **p < 0.01; ***p < 0.001.

The effect of food simulants and the combination of acetone and methanol was also tested for potential background effects or interference with the in vitro micronucleus assay conducted on HepG2 and Caco‐2 cell lines. The subjects were tested for three different treatments as required by OECD technical guideline 487. Food simulants did not introduce significant effects on the HepG2 cell line, as shown in Figure 9a. The same was observed for the Caco‐2 cell line in Figure 9b. Food simulant D1 introduced a small, nonsignificant increase in the frequency of micronuclei in the Caco‐2 cell line following long treatment.

The aforementioned conditions for the food simulants and organic solvents fulfill the preliminary requirements of OECD technical guidelines 471 for the bacterial reverse mutation assay (Ames test) and 487 for the in vitro micronucleus assay regarding the absence of cytotoxicity and the formation of homogeneous exposure conditions. Additionally, the food simulants do not interfere with the analysis of mutagenic and genotoxic effects via the bacterial reverse mutation assay and in vitro micronucleus assay. Consequently, the proposed conditions are suitable for assessing such effects via the assays required by EFSA.

## 4 CONCLUSION

Commission Regulation (EU) 10/2011 outlines specific testing conditions for evaluating the safety of plastic FCMs. This study provides guidance on using these conditions to prepare samples compatible with in vitro biological testing. The proposed maximum concentrations of food simulants and the additional preparatory step for food simulant E help avoid background toxicity that could influence the safety assessment of FCMs, potentially leading to an overestimation of toxicity. Additionally, this study proposes compatible assays for assessing hepatotoxic effects and colon toxicity using HepG2 and Caco‐2 cell lines. The proposed types and concentrations of solvents do not interfere with the performance of the bacterial reverse mutation assay and the in vitro micronucleus assay. Therefore, these methods are compatible with the mandatory testing for FCMs required by the EFSA.

## AUTHOR CONTRIBUTIONS

**Athanasios Kourkopoulos**: Conceptualization; methodology; software; data curation; investigation; validation; formal analysis; visualization; project administration; resources; writing—original draft. **Dick T. H. M. Sijm**: Conceptualization; supervision; funding acquisition; project administration; writing—review and editing. **Janneke Geerken**: Investigation. **Misha F. Vrolijk**: Conceptualization; supervision; funding acquisition; project administration; writing—review and editing.

## CONFLICT OF INTEREST STATEMENT

The authors declare that there are no conflicts of interest.
